# Supplementary material for: Practice regarding tuberculosis care among physicians at private facilities: A cross-sectional study from Vietnam
Source: PLoS One. 2023 Apr 27;18(4):e0284603. doi: 10.1371/journal.pone.0284603 (PMC10138252; doi:10.1371/journal.pone.0284603)
Supplement: S1 Appendix — (DOCX) [file pone.0284603.s001.docx]

**Tables**

Table S1. Factors associated with knowledge regarding TB (n = 232).

| Factors | Coefficient (95%CI) |
| --- | --- |
| Female | -0.120 (-0.317, 0.076) |
| Duration in current position | **0.019 (0.007, 0.032)** |
| Highest degree (reference group: Medical doctor) |  |
| Specialist level 1/Master | **-0.398 (-0.620, -0.175)** |
| Specialist level 2/PhD | **-0.631 (-1.117, -0.145)** |
| Speciality (reference group: Primary care) |  |
| Internal medicine | **-0.236 (-0.495, 0.024)** |
| Pulmonary medicine | **-0.287 (-0.562, -0.012)** |
| Family medicine | **-0.543 (-0.945, -0.142)** |
| Having prior TB training | **1.009 (0.764, 1.254)** |

**Abbreviations**: TB, tuberculosis.

Coefficients in bold are statistically significant.

Table S2. Factors associated with attitude (n = 232),

| Factors | Stigma towards TB | Disbelief in the TB program | Motivation for TB care |
| --- | --- | --- | --- |
| Female | -0.055 (-0.281, 0.172) | -0.175 (-0.402, 0.053) | 0.044 (-0.177, 0.265) |
| Duration in the current position | -0.010 (-0.025, 0.005) | 0.003 (-0.012, 0.018) | 0.014 (-0.000, 0.029) |
| Highest degree (reference group: Medical doctor) |  |  |  |
| Specialist level 1/Master | **0.388 (0.125, 0.651)** | 0.184 (-0.080, 0.449) | **-0.332 (-0.588, -0.075)** |
| Specialist level 2/PhD | **0.999 (0.433, 1.566)** | 0.346 (-0.224, 0.915) | -0.417 (-0.970, 0.135) |
| Speciality (reference group: Primary care) |  |  |  |
| Internal medicine | 0.211 (-0.089, 0.512) | 0.185 (-0.117, 0.486) | -0.121 (-0.413, 0.172) |
| Pulmonary medicine | 0.253 (-0.061, 0.567) | 0.119 (-0.197, 0.434) | -0.196 (-0.502, 0.110) |
| Family medicine | 0.433 (-0.035, 0.900) | 0.165 (-0.304, 0.635) | -0.430 (-0.886, 0.025) |
| Knowledge | **-0.344 (-0.478, -0.211)** | **-0.408 (-0.542, -0.274)** | **0.321 (0.191, 0.451)** |

**Abbreviations**: TB, tuberculosis.

Coefficients in bold are statistically significant.
